# The critical role of neutrophil-endothelial cell interactions in sepsis: new synergistic approaches employing organ-on-chip, omics, immune cell phenotyping and *in silico* modeling to identify new therapeutics

**DOI:** 10.3389/fcimb.2023.1274842

**Published:** 2024-01-08

**Authors:** Dan Liu, Jordan C. Langston, Balabhaskar Prabhakarpandian, Mohammad F. Kiani, Laurie E. Kilpatrick

**Affiliations:** ^1^ Department of Bioengineering, Temple University, Philadelphia, PA, United States; ^2^ Biomedical and Data Sciences Division, CFD Research Corporation, Huntsville, AL, United States; ^3^ Department of Mechanical Engineering, Temple University, Philadelphia, PA, United States; ^4^ Department of Radiation Oncology, Lewis Katz School of Medicine, Temple University, Philadelphia, PA, United States; ^5^ Center for Inflammation and Lung Research, Department of Microbiology, Immunology and Inflammation, Lewis Katz School of Medicine, Temple University, Philadelphia, PA, United States

**Keywords:** biomimetic microfluidic assay, endothelial cells, inflammation, *in silico* modeling, neutrophils, omics, organ-on-chip, sepsis

## Abstract

Sepsis is a global health concern accounting for more than 1 in 5 deaths worldwide. Sepsis is now defined as life-threatening organ dysfunction caused by a dysregulated host response to infection. Sepsis can develop from bacterial (gram negative or gram positive), fungal or viral (such as COVID) infections. However, therapeutics developed in animal models and traditional *in vitro* sepsis models have had little success in clinical trials, as these models have failed to fully replicate the underlying pathophysiology and heterogeneity of the disease. The current understanding is that the host response to sepsis is highly diverse among patients, and this heterogeneity impacts immune function and response to infection. Phenotyping immune function and classifying sepsis patients into specific endotypes is needed to develop a personalized treatment approach. Neutrophil-endothelium interactions play a critical role in sepsis progression, and increased neutrophil influx and endothelial barrier disruption have important roles in the early course of organ damage. Understanding the mechanism of neutrophil-endothelium interactions and how immune function impacts this interaction can help us better manage the disease and lead to the discovery of new diagnostic and prognosis tools for effective treatments. In this review, we will discuss the latest research exploring how *in silico* modeling of a synergistic combination of new organ-on-chip models incorporating human cells/tissue, omics analysis and clinical data from sepsis patients will allow us to identify relevant signaling pathways and characterize specific immune phenotypes in patients. Emerging technologies such as machine learning can then be leveraged to identify druggable therapeutic targets and relate them to immune phenotypes and underlying infectious agents. This synergistic approach can lead to the development of new therapeutics and the identification of FDA approved drugs that can be repurposed for the treatment of sepsis.

## Introduction

Sepsis is a critical healthcare problem. According to a meta-analysis of literature by the World Health Organization (WHO), in 2017 approximately 48.9 million cases of sepsis were identified worldwide, accounting for 11 million sepsis-related deaths per year (World Health Organization, 2023). An estimated 20 million children younger than 5 years developed sepsis, resulting in 2.9 million global deaths in the same year. In the U.S., 1.7 million sepsis cases are reported annually, resulting in more than 270,000 deaths each year (Rhee et al., 2017) and is a leading cause of death in hospitals (Singer et al., 2016; Donnelly et al., 2017). Under the Sepsis-3 definition, sepsis is now defined as life-threatening organ dysfunction caused by a dysregulated host response to infection (Singer et al., 2016; Donnelly et al., 2017). While neutrophils are critical to host defense, neutrophil dysregulation in sepsis plays a critical role in sepsis-induced organ failure through interactions with the vascular endothelium resulting in endothelial cell (EC) damage, increased vascular barrier permeability, and enhanced neutrophil trafficking into vital organs (Guimarães-Costa et al., 2014; Lang et al., 2014; Jiao et al., 2020; Iba et al., 2022; Rosales, 2020; Sakuma et al., 2022). This neutrophil dysregulation and neutrophil-endothelial cell interactions in sepsis can lead to extravascular tissue damage and septic shock resulting in multiple organ dysfunction syndrome (MODS) and death (Kell and Pretorius, 2018).

Sepsis is a complex and multifaceted medical condition, and its manifestations can vary considerably among patients. It is not a uniform disease but rather a syndrome that results from the body’s overwhelming response to infection. The clinical presentation of sepsis can range from mild to severe, with some individuals experiencing subtle symptoms while others face life-threatening consequences, such as organ dysfunction and septic shock (Singer et al., 2016; Seymour et al., 2019). Sepsis is also known for its remarkable heterogeneity in clinical manifestations, and this diversity is often influenced by both the source of infection and the unique immune response of the affected individual. For example, sepsis originating from a respiratory infection, such as pneumonia, may present with distinctive clinical features as compared to sepsis triggered by a urinary tract infection (Rudd et al., 2020). The former may involve respiratory distress, high fever, and lung-specific complications, while the latter might primarily manifest as urinary symptoms and lower abdominal pain. These variations in sepsis presentation underscore the significance of personalized and targeted treatment approaches to address the diverse clinical trajectories of this life-threatening condition. Recent research has focused on the heterogeneity of sepsis, emphasizing the necessity of understanding the source-specific and patient-specific aspects of sepsis (Seymour et al., 2019).

**Figure 1:**
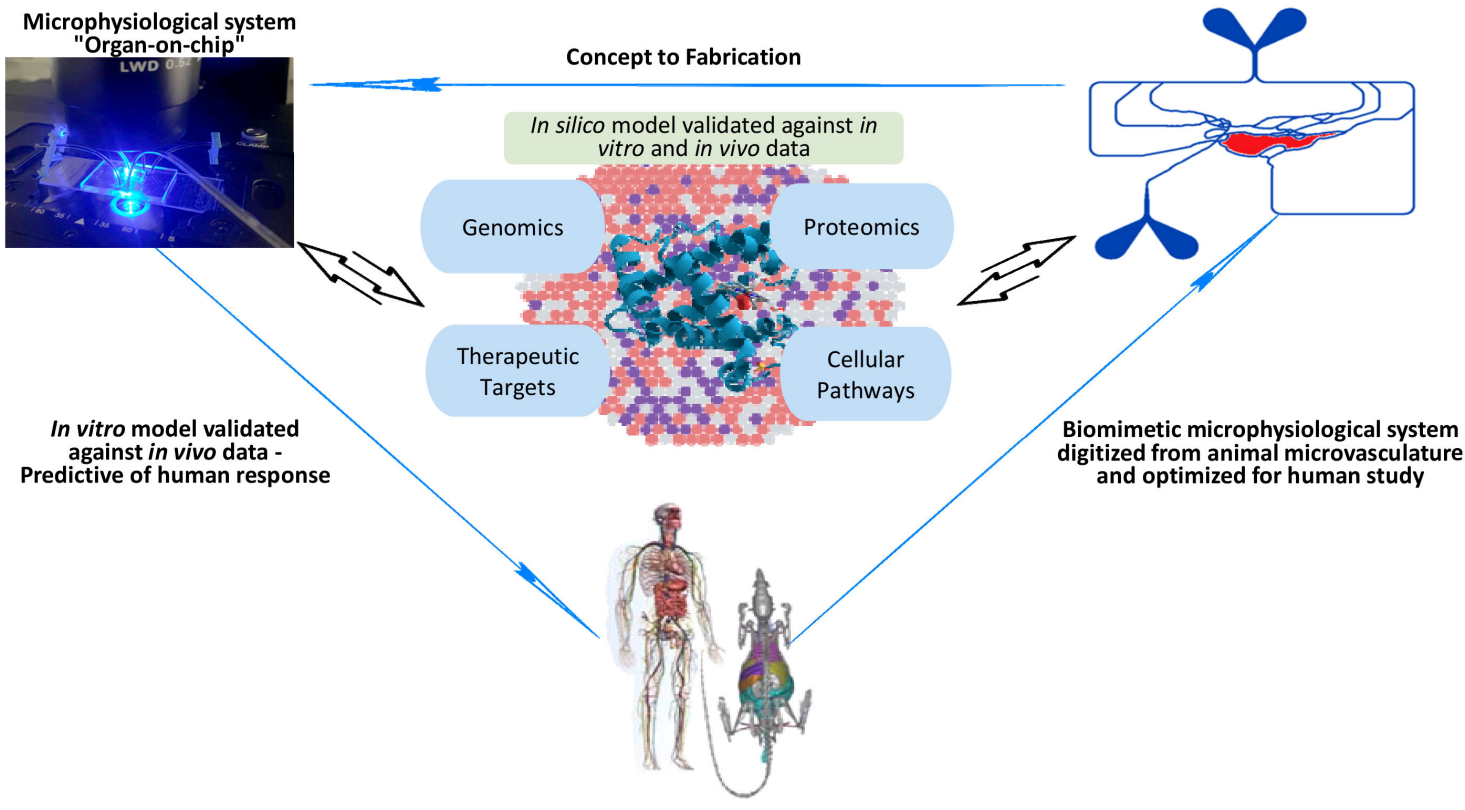
An illustration of how emerging tools such as biomimetic microphysiological systems (i.e., organ-on-chip), omics, *in silico* models and machine learning are providing promising avenues for better understanding and developing more effective, personalized options for treating sepsis.

To date, therapeutic approaches for the treatment of sepsis are largely supportive, but there are no specific drugs available despite promising preclinical studies in rodent models (Williams and Chambers, 2014; Jarczak et al., 2021; Niederman et al., 2021). Current treatment consists of antibiotic therapy for the underlying infection and supportive care through the administration of fluids and vasopressors (Seitz et al., 2022). Kidney dialysis and mechanical ventilation are often used to support organ failure as the disease progresses. Multiple drugs which have shown promise in preclinical models have failed in large randomized clinical trials to demonstrate a significant reduction in mortality (Osborn, 2017). This absence of clinical translation is due to a host of factors, including the incorporation of rodent models that fail at emulating the complete clinical situation that is present in humans (e.g., age, sex, demographics, comorbidities etc.) and the varied composition of systemic leukocytes in both species (Rudd et al., 2020; Iregbu et al., 2022; World Health Organization, 2017). Recent clinical trials have examined the impact of anti-cytokine receptor therapies such as anakinra (an IL-1 receptor antagonist) to mitigate the inflammatory response (Shakoory et al., 2016), and the consideration of using DNase (such as DNase-I) in addressing sepsis-associated formation of neutrophil extracellular traps (NETs), thereby highlighting the multifaceted approach required for effective sepsis treatment (Singer et al., 2016; Donnelly et al., 2017). Unfortunately, none of these treatments demonstrate targeted efficacy against sepsis. Numerous reports have highlighted the heterogeneous nature of sepsis (Scicluna et al., 2017; Fenner et al., 2021; Yang et al., 2021b; Langston et al., 2023), confirming the unlikelihood that a one-size-fits-all treatment will be appropriate for every septic patient. Thus, the lack of therapeutic options for the treatment of sepsis underscores the urgent need for further research and innovation to develop novel therapeutic interventions that can address the complexities of this life-threatening condition (Langston et al., 2023). Emerging tools such as microphysiological assays (i.e., organ-on-chip) (Kilpatrick and Kiani, 2020; Yang et al., 2021a), omics, *in silico* models and machine learning (Giannini et al., 2019; Stolarski et al., 2022; Honoré et al., 2023; Pan et al., 2023) are providing promising avenues for developing effective, personalized options for treating sepsis (**Figure 1**).

In this review, we will examine critical aspects of neutrophil-ECs interactions during the evolution and progression of sepsis, highlighting the difficulties in development of effective therapeutic approaches. We will discuss the emergence of innovative *in vitro* models that mimic the complex interplay between neutrophils and ECs and the use of these models in classifying sepsis patients and testing potential therapeutics. We will focus on the importance of immune phenotyping and sepsis endotyping to gain important insight into the heterogenous nature of the disease. We will discuss the importance of omics in identifying important cellular signaling cascades involved in sepsis progression. Lastly, we will discuss promising applications of *in silico* models and machine learning algorithms, offering new avenues for enhancing our comprehension of sepsis.

## Neutrophil activation during sepsis

Neutrophils are key cells of the human innate immune system and critical elements of host defense against pathogens. Neutrophils identify infectious agents through pattern recognition receptors (PRRs), such as Toll-like receptors, on the cell surface that recognize a wide variety of PAMPs and DAMPS that are released by invading pathogens and damaged tissue (Iba and Ogura, 2018). Upon activation, neutrophils rapidly traffic to sites of infection or inflammation (Ley et al., 2007). Neutrophils clear pathogens through phagocytosis, degranulation and the release of proteases and the production of reactive oxygen species (ROS), which can kill pathogens directly by causing oxidative damage (Perobelli et al., 2015; Glémain et al., 2022; Rosales, 2018). Neutrophils possess numerous types of granules within their cytoplasm that are sequentially released upon stimulation and degranulation. Neutrophils contain primary (azurophilic), secondary (specific) and tertiary (gelatinase) granules, as well as secretory vesicles encompassing a repertoire of more than 300 proteins involved in cell adhesion, migration and bactericidal activities (Fox et al., 2010). A number of potent proteases are located in these granules including myeloperoxidase (MPO), neutrophil elastase and cathepsin G (Borregaard, 2010). The release of these proteases is tightly regulated to minimize host tissue damage, but during sepsis and immune dysregulation, the release of these granule contents can damage ECs and host tissue. Neutrophils can also immobilize and kill pathogens through the release of NETs (Perobelli et al., 2015; Herrero-Cervera et al., 2022; Rosales et al., 2023). NETs are composed of extruded strands of nuclear material (such as DNA) which form a web-like structure that is composed of decondensed chromatin fibers decorated with antimicrobial enzymes released from granules, such as neutrophil elastase, MPO and cathepsin, as well as nuclear proteins (Perobelli et al., 2015; Döring et al., 2017; Jiao et al., 2020). NETs can be a double-edged sword; they are critical for microbicidal activity, but uncontrolled NETs release during sepsis can also exacerbate inflammation, damage ECs, and cause tissue injury (Chang, 2019; Glémain et al., 2022).

Thus, neutrophil activation plays a pivotal role during sepsis, with a complex interplay between various cellular and molecular mechanisms. The activation of neutrophils leads to a cascade of events, including adhesion, migration, degranulation, and the formation of NETs. However, dysregulated neutrophil activation contributes to EC damage and organ dysfunction.

## Endothelial cell activation during sepsis

Under normal conditions, ECs act as an important regulator of hemodynamic balance throughout the body. ECs possess a remarkable ability to sense and react to various extracellular stimuli originating from the microenvironment (Yang et al., 2021b). These stimuli encompass both biomechanical factors (e.g., shear forces, pressure, and cyclical strain) as well as biochemical signals (e.g., growth factors, hormones, cytokines, chemokines, nitric oxide, oxygen and reactive oxygen species) (Rosales, 2018). The ability to sense hemodynamic stimuli allows ECs to quickly respond to microenvironmental changes in the blood vessel environment, such as sensing the pro-inflammatory cytokines released by neutrophils during sepsis (Iba et al., 2022). Notably, in response to these stimuli, ECs exhibit distinct phenotypes characterized by spatial and temporal tissue-specific heterogeneity (Aird, 2012).

The diverse morphological characteristics of endothelium are directly related to their functional heterogeneity (Yang et al., 2021b). For example, within arteries and arterioles, ECs align and elongate in parallel to the direction of blood flow, playing a crucial role in regulating vascular tone (Yang et al., 2021b). In contrast, ECs in veins and venules typically assume a polygonal shape rather than an elongated shape, lacking specific orientation (Yang et al., 2021b). Moreover, venous ECs tend to have higher permeability compared to arterial ECs (Sriram et al., 2015).Notably, post-capillary venules serve as primary sites for leukocyte extravasation during inflammation and sepsis, facilitating the movement of immune cells from the bloodstream into the surrounding tissues (Chi et al., 2003). These contrasting characteristics of ECs are closely linked to variances in hemodynamic environments and the functional properties of the vessels they line (Young and Simmons, 2010).

Endothelial cells (ECs) also exhibit remarkable heterogeneity across different organs, and their distinct phenotypic profiles can play varying roles in sepsis. For instance, lung microvascular ECs are characterized by high levels of tissue factor expression, which contributes to the initiation of coagulation pathways during sepsis (Wang et al., 2008). In contrast, hepatic ECs possess unique functional features, including the clearance of endotoxins and bacterial products where the EC structure and alignment are discontinuous, allowing the exchange of large solutes between plasma and intestinal environment (Shetty et al., 2018). On the other hand, kidney EC exhibits a fenestrated alignment that features intracellular pores with a diaphragm that penetrate the endothelium layer, which warrants the rapid exchange of water (Yang et al., 2021b). This specific EC alignment in kidney plays a crucial role in sepsis by potentially enabling enhanced vascular permeability and leukocyte trafficking, thereby contributing to the pathophysiological processes and kidney dysfunction during sepsis progression. These organ-specific variation in EC function during sepsis is critical for understanding the differential responses observed in discrete organs. At the molecular level, these organ-specific disparities can be attributed to differences in the expression of adhesion molecules, cytokine receptors, and signaling pathways. For instance, the lung ECs express higher levels of vascular cell adhesion molecule 1 (VCAM-1) and intercellular adhesion molecule 1 (ICAM-1), which are involved in leukocyte adhesion and recruitment (Ley et al., 2007). Liver ECs, on the other hand, express receptors for bacterial toxins, enabling them to actively participate in detoxification processes (Dejager et al., 2011). Vascular permeability is regulated by tight and adherens junctions between ECs, therefore, the disassembly of occludin, claudin-5 and VE-cadherin from tight junctions can be used as an indicator of increased vascular permeability (Kondo et al., 2009). Aslan et al. reported that baseline expression levels of occludin, claudin-5, and VE-cadherin markedly differed between kidney and lung, with claudin-5 and VE-cadherin being highly expressed in the lung, and occludin being highly expressed in the kidney during sepsis in mice and humans, indicating an organ-specific molecular manifestation of ECs (Aslan et al., 2017). This intricate web of organ-specific heterogeneity in EC function during sepsis not only enhances our understanding of the diverse organ responses but also underscores the pivotal role of these cells in shaping the outcomes of this complex and life-threatening condition.

The endothelial glycocalyx (eGC) is a protective layer on the cell surface of vascular ECs (Fatmi et al., 2022) that plays a critical role during inflammation. This glycoprotein layer has an intricate architecture consisting of different components integrating either plasma- or endothelium-derived soluble molecules, including syndecan-1 (SDC-1), Heparan sulphaste (HS), Hyaluronan (HA), chondroitin sulphate (CS), and other cell adhesion molecules (CAMs) such as VCAM-1 and ICAM-1 (Puchwein-Schwepcke et al., 2021; Fatmi et al., 2022). These important surface molecules play critical roles during the leukocyte adhesion cascade in sepsis (Ley et al., 2007; Iba and Ogura, 2018; Iba et al., 2022). Activated neutrophils can damage the protective EC glycocalyx leading to increased vascular barrier permeability and neutrophil trafficking into vital organs (Chang, 2019; Iba et al., 2022).

**Figure 2:**
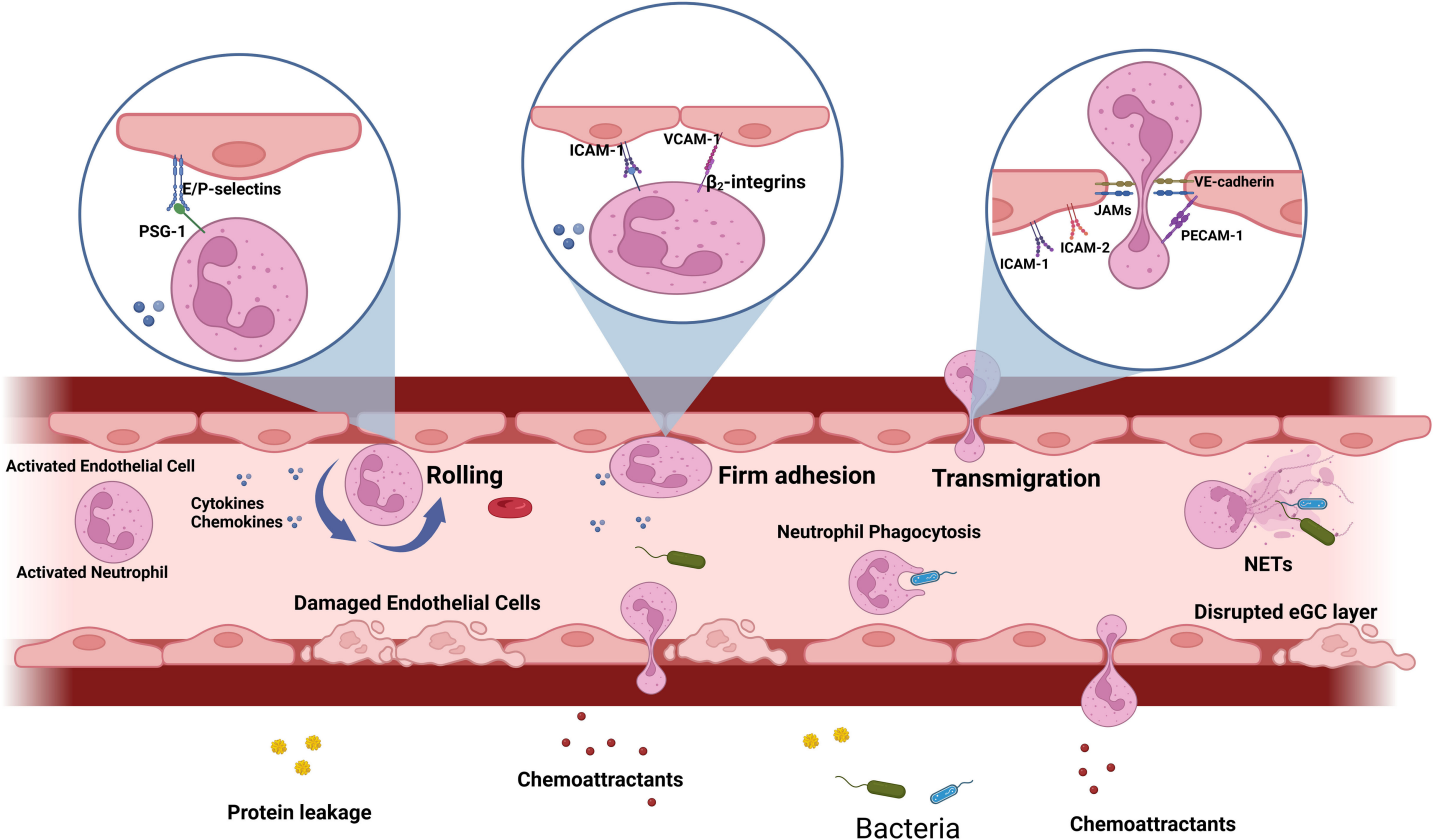
Leukocyte adhesion cascade during sepsis: During sepsis, both PAMPs and DAMPs trigger the activation of neutrophils and endothelial cells, prompting the production of cytokines and chemoattractants. Consequently, neutrophils display surface molecules that interact with adhesion molecules expressed by activated endothelium. The initial rolling step involves interactions between E/P-selectin and their ligands, such as PSGL-1, which slows down the neutrophil. Subsequently, firm adhesion is mediated by endothelial adhesion molecules like ICAM-1, ICAM-2, and VCAM-1, interacting with neutrophil ligands, such as β2 integrins. In response to chemoattractants, adhered neutrophils migrate through endothelial junctions, facilitated by PECAM-1, VE-cadherin and JAMs. Concurrently, activated neutrophils release cytokines, reactive oxygen species (ROS), and proteases, or undergo the formation of neutrophil extracellular traps (NETs). During sepsis, the endothelial glycocalyx (eGC) is degraded, endothelial cell tight junctions are damaged, and there is an increase in endothelial cell apoptosis, ultimately leading to compromised barrier function and increased permeability.

ECs are tightly connected through junctional adhesion molecules (JAMs) including JAM-A, -B, -C and endothelial cell-selective adhesion molecules (ESAM) which are crucial for maintaining the endothelial junctional complexes that regulate vascular transmigration and permeability (Woodfin et al., 2011; Amalakuhan et al., 2016). During sepsis, these junctional functions are disrupted and become dysregulated, leading to increased vascular permeability and, fluid leakage into the surrounding tissue and edema, causing further organ damage (**Figure 2**) (Nourshargh and Alon, 2014; Uchimido et al., 2019; Duong and Vestweber, 2020). Other junctional components include platelet endothelial cell adhesion molecule (PECAM)-1 and nectin; the disruption of these components also contribute to the uncontrolled neutrophil influx (Duong and Vestweber, 2020).

In summary, ECs play a pivotal role in maintaining vascular health and homeostasis, and their injury can trigger a cascade of events leading to tissue and organ dysfunction. EC injury can occur due to various insults, such as inflammation, oxidative stress or mechanical damage. At the molecular level, the loss of EC integrity disrupts the endothelial barrier, exposing underlying tissues to a pro-inflammatory environment. Moreover, injured ECs release vasoactive molecules, such as endothelin-1, leading to vasoconstriction and impaired blood flow (Aird, 2012). Disruption of the endothelial barrier can also result in increased vascular permeability, allowing the leakage of proteins and immune cells into the interstitium, further exacerbating inflammation (Chistiakov et al., 2015). Over time, these processes can lead to chronic inflammation, tissue damage, and ultimately, organ dysfunction. Understanding the molecular mechanisms underlying EC injury and dysfunction is crucial for developing targeted therapeutic interventions to mitigate the consequences of endothelial damage (Libby et al., 2011).

## Neutrophil-endothelium interaction during sepsis

Septic patients often die from MODS, in part caused by the dysregulated neutrophil influx into tissues leading to organ damage (Yang et al., 2021a). During infection, pathogen-associated molecular patterns (PAMPs) and damage-associated molecular patterns (DAMPs) are released and activate the innate immune system. This activation results in the release of cytokines, chemokines and other proinflammatory mediators which can activate neutrophils and endothelial cells (ECs) leading to neutrophil-EC interaction.

The leukocyte adhesion cascade is a process by which leukocytes, such as neutrophils, roll, firmly adhere and then migrate through activated ECs (Ley et al., 2007; Futosi et al., 2013; Tecchio et al., 2014; Rosales, 2018; Rosales, 2020). This process is mediated on ECs by pro-adhesive and other effectors molecules through the Nuclear factor-kappa B (NF-κB) signaling pathway (Kojok et al., 2018). Pattern recognition receptors (PRRs) are widely expressed on neutrophils and ECs (Iba and Ogura, 2018). When these receptors interact with PAMPs and DAMPs, cytokines [e.g., Tumor necrosis factor-alpha (TNF-α) and Interleukin-1 Beta (IL-1β)] and chemokines [e.g., Interleukin-8 (IL-8) and Monocyte chemoattractant protein 1 (MCP-1)] are synthesized and released into the bloodstream (Zhu et al., 2017; Kojok et al., 2018; Wang et al., 2018; Georgescu et al., 2020). These pro-inflammatory cytokines disrupt the integrity of the endothelial glycocalyx (eGC) surface layer, exposing adhesion molecules, such as VCAM-1, ICAM-1 and E-selectin and result in increased number of neutrophils rolling, firmly adhering and transmigrating through the ECs (Schmidt et al., 2012; Yang et al., 2021b). As sepsis advances, VCAM-1 and ICAM-1, which are expressed in large quantities on the activated EC surface of septic patients, are also cleaved and released into the circulation (Amalakuhan et al., 2016). **Figure 2** illustrates the key steps of the leukocyte adhesion cascade process during the onset of sepsis and highlights the unique characteristics of neutrophils and ECs that allow them to interact effectively during sepsis.

Better understanding of neutrophil-endothelium interaction can help identify potential therapeutic targets to prevent the development of organ dysfunction in sepsis.

## Sepsis manifestations and existing models for sepsis research and their limitations

Animal models and *in vitro* models employing cell culture and organoids, have proven invaluable for unraveling some of the intricacies of sepsis pathophysiology. The classical approach in exploring the mechanisms of inflammatory diseases involves the use of murine models. However, the lack of alignment between murine models and human septic manifestations is a growing concern (Soroush et al., 2020). Additionally, the significant phenotypic variation observed among various types of endothelial cells has raised questions about the translatability of findings from these models to human diseases (Seok et al., 2013; Sznajder et al., 2013). Consequently, a notable limitation of murine models is the potential for a given therapeutic intervention to yield distinct outcomes in mice compared to humans (Seok et al., 2013; Sznajder et al., 2013). These concerns gain support from recent research employing bulk and single-cell transcriptomics to delineate innate immune responses. This work has demonstrated substantial interspecies variations in the expression of cytokines, chemokines, and their corresponding receptors (Hagai et al., 2018). While rodent models remain a valuable tool in sepsis research, an international panel of experts has emphasized the pressing need for models that more realistically recapitulate human disease, acknowledging the significant limitations of murine models (Zingarelli et al., 2019). Hence, there exists a considerable demand for the development of “*in vitro* reconstitution of disease-related cell types or tissues” employing human cells to investigate human inflammatory diseases more effectively (Kilpatrick and Kiani, 2020; Soroush et al., 2020; Yang et al., 2021a). Reconstruction of more *in vivo* human relevant models could bridge the gap between traditional murine models and the intricacies of human inflammatory disease, potentially advancing our understanding and therapeutic strategies in this field.

Traditional cell culture models have been used to study molecular and cellular responses to septic insults. These models typically involve specific cell types, such as macrophages or endothelial cells, and exposing them to PAMPs, such as endotoxin, or proinflammatory cytokines to mimic septic conditions. These *in vitro* studies allow for the investigation of host responses, such as the activation of PRRs which activate NF-κB and other transcription factors to produce proinflammatory mediators such as cytokines and chemokines. (Ploppa et al., 2012; Mai et al., 2013; Wicherska-pawłowska et al., 2021). Despite their utility and ease of use, cell culture models have significant limitations. They often lack the intricate three-dimensional tissue architecture seen *in vivo*, which is crucial for studying cell-cell interactions. Moreover, these models may not fully replicate the complexity of the immune response in sepsis, as they often lack multiple cell types important for cell-cell communication (Chistiakov et al., 2015). More importantly, static cell culture models do not take in to account the impact of physiologically relevant flow conditions which is a critical parameter in cell activation and signaling and needed to model *in vivo* conditions. Organoids represent another promising approach for *in vitro* studies of sepsis. These miniature 3D organ-like structures are developed by culturing organ-specific cells, such as intestinal tissue (Siwczak et al., 2021), lung tissue (Bosáková et al., 2022), or brain tissue (Lim et al., 2022) under conditions that promote self-organization and differentiation. Recent advancements have allowed researchers to create lung, liver, and intestinal organoids to mimic *in vivo* inflammation more realistically. These models provide valuable insights into the tissue-specific molecular responses during inflammatory conditions (Lim et al., 2022), including the dysregulation of tight junction proteins, altered metabolic pathways and the release of proinflammatory cytokines (Lee et al., 2014). However, organoids, too, have their limitations. They may not fully capture the systemic effects of sepsis, given their focus on individual organs. Additionally, their utility is currently restricted by challenges in scaling up for high-throughput screening and maintaining long-term viability. Last but not least, the vascular network of organoids is hard to reconstruct *in vitro*, therefore, these organoid models often lack realistic blood flow representation as well.

In summary, organoids offer insights into tissue-specific responses while murine animal models could provide a more translational understanding to human *in vivo* conditions. These models enhance our understanding of sepsis pathophysiology, but researchers must be mindful of their limitations, such as the lack of tissue complexity in cell cultures, the limited systemic perspective of organoids, and the significant limitations of animal models. Addressing these challenges is essential for optimizing the translational potential of these models in sepsis research.

## Emerging *in vitro* models – the promise of the organ-on-chip technology

As discussed previously, excessive neutrophil infiltration into tissues causes organ damage, often resulting in organ failure and death during sepsis. Employing screening models that mimic neutrophil-endothelium interactions under inflammatory conditions have become important tools for developing precision therapeutics.

Development of “organ-on-chip” assays, also known as microphysiological systems or 3-D biomimetic microfluidic assays, offer a number of advantages including the ability to observe and measure in real time neutrophil-endothelial cell interactions such as chemotaxis, upregulation of adhesion molecules and changes in response to various therapeutic agents (Ren et al., 2022; Man et al., 2023). The ability to use primary human cells in microphysiological systems increases their clinical relevance. For example, Muldur et al. utilized a microfluidic system to examine the distinct effects of different types of Complement 5 (C5) cleavage inhibitors on human neutrophils activities including anti-microbial functions, chemotaxis and swarming (Muldur et al., 2021). Zhang et al. developed a microfluidic system to measure affinity separation to capture the concentration of Cluster of differentiation (CD64)+ cells and provided evidence that neutrophil CD64 expression is upregulated in sepsis patients (Zhang et al., 2018).

The formation of NETs is a critical step in antimicrobial defense through the immobilization of pathogens (Jiao et al., 2020). Microfluidic assays have been used to investigate NETs. For example, Sakuma et al. developed a microfluidic assay to capture and measure NETs in a drop of human blood using immunostaining and fluorescence microscopy (Sakuma et al., 2022). In this system, using as little as 10 uL of blood, without neutrophil or plasma separation, the assay consistently measures the sum of intact and degraded NETs (Sakuma et al., 2022). “Open-well” barrier based microfluidic designs could potentially replicate the compartmentalization found *in vivo* (Byrne et al., 2017). In these assays, primary human cells can be seeded directly onto the membrane that physically separates the different compartments while permitting soluble factor communication between the cell populations (Byrne et al., 2017). The conventional transwell assay design has been used to study the blood-brain barrier (Stone et al., 2019), alveolar-capillary interface (Janga et al., 2018) and glomerular barrier (Moriyama et al., 2017). However, these transwell model designs lack fluid flow, an important component in the *in vivo* microenvironments. In order to address this constraint, Mehran et al. integrated laminar flow into the open-well model concept and developed an open-well modular system (m-uSiM) utilizing a silicon membrane (thickness=100nm, porosity=15%), establishing a human endothelial cell layer in the device that could be visualized under a microscope (Mansouri et al., 2022).

Aside from the visualization of the cell-cell interaction, another important advantage of these assays is the ability to study neutrophil behavior and functions in real-time. For example, studies have shown that when using biomimetic microfluidic assays, neutrophil migration (Boribong et al., 2019) and NETosis (Petchakup et al., 2023) can be observed. Furthermore, biomimetic microfluidic assays can be used to observe the process of neutrophils eliminating pathogens such as fungal clusters (Alex et al., 2020) and bacteria (Ellett et al., 2019) in real-time.

**Figure 3:**
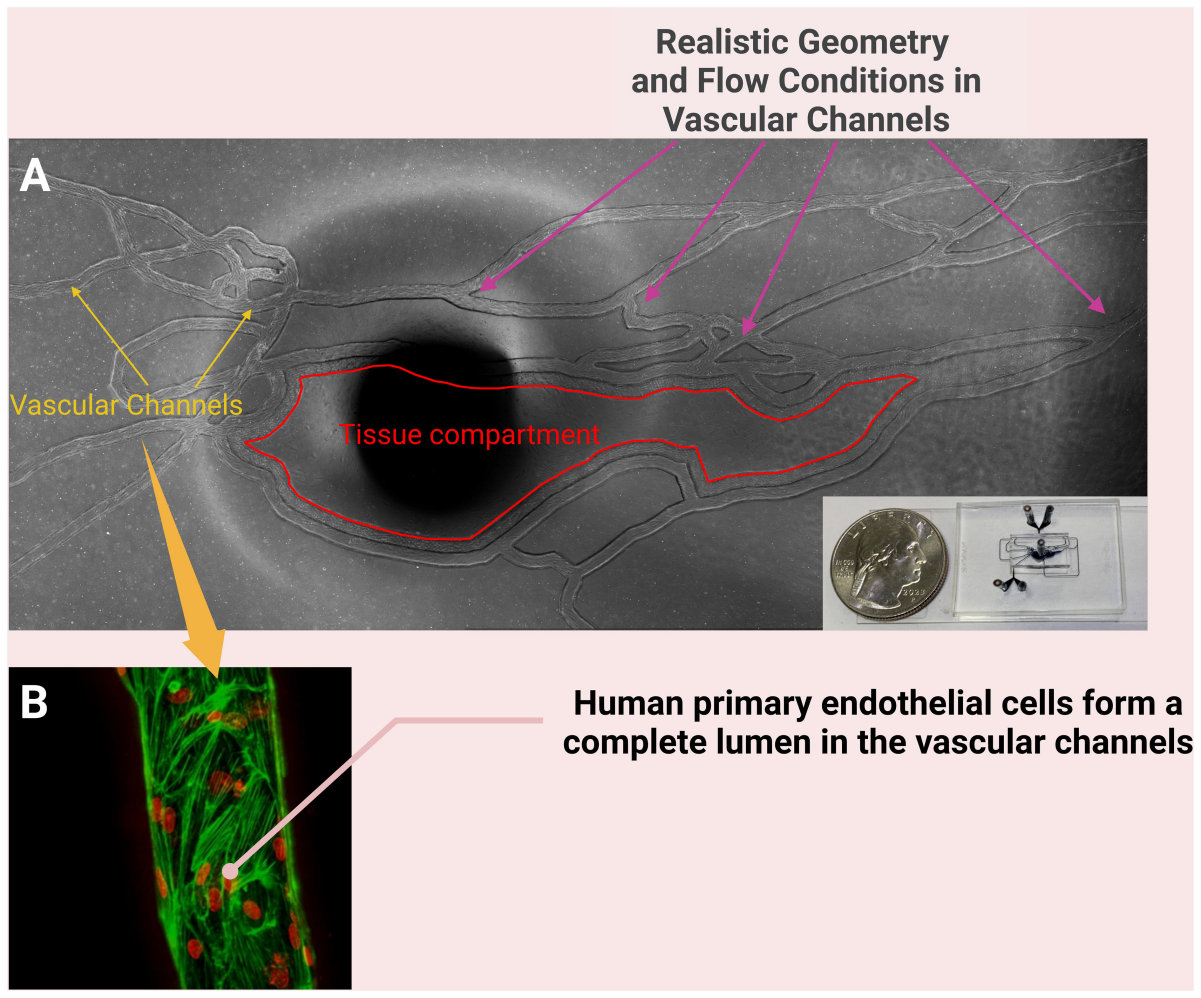
A biomimetic microfluidic assay that reproduces complete microvascular networks on a chip to study the effect of inflammation and sepsis on endothelial permeability and of neutrophil-endothelial cell interaction **(A)**; the dark spot in the middle of **(A)** is the port for connecting tubing to the tissue compartment. The vascular channels of this system are cultured with primary human endothelial cells **(B)** and the neutrophil-endothelial cell interaction can be studied under physiologically relevant shear stress levels in the presence of chemoattractants such as fMLP in the tissue compartment. [**(B)** reproduced with permission (Soroush et al., 2020)].

Many of the microfluidic devices used for studies of the inflammatory response do not completely reproduce the geometry and structure of microvascular networks observed *in vivo*. To better model the neutrophil-endothelium interaction during sepsis using primary human cells in a more realistic environment, our group has developed a biomimetic microfluidic assay that reproduces complete microvascular networks on a chip to study the effect of inflammation and sepsis on endothelial permeability and neutrophil-endothelial cell interaction (Yang et al., 2021a). This system can be used to observe the entire leukocyte adhesion cascade including rolling, firm adhesion, spreading and migration of neutrophils into the tissue compartment (**Figure 3**), mediated by the presence of chemoattractants such as fMLP in the tissue compartment. Neutrophil adhesion and migration could then be measured under physiologically relevant shear stress levels in response to, for example, pro-inflammatory cytokines (Soroush et al., 2016). We have used this microfluidic assay to show that a novel Protein Kinase C-delta (PKCδ) inhibitor not only reduces neutrophil adhesion to and migration across ECs but also decreases the expression of ICAM-1 and other adhesion molecules on ECs during the onset of inflammation (Soroush et al., 2016; Kilpatrick and Kiani, 2020; Soroush et al., 2020). The findings from our microfluidic system have been validated against *in vivo* observations in sepsis animal models (Soroush et al., 2016). In a rodent model of sepsis, administration of the PKCδ inhibitor significantly decreased sepsis-induced neutrophil influx into the lungs and kidneys (Liverani et al., 2020). Furthermore, PKCδ inhibition was lung protective and decreased sepsis-induced VCAM-1 and ICAM-1 endothelial expression (Mondrinos et al., 2014).

ECs are heterogeneous, in part due to organ type, and it is therefore critical to develop organ-on-chip assays that realistically represent the conditions in each organ. Our group, for example, has employed a novel blood-brain-barrier on-a-chip (B^3^C) microfluidic assay to study the role of PKCδ in mediating human brain microvascular endothelial cell (HBMVEC) permeability, junctional protein expression and leukocyte adhesion and migration (Tang et al., 2018). Our findings indicated that TNF-α activates PKCδ and its translocation in HBMVEC (Tang et al., 2018). Inhibition of PKCδ significantly reduced TNF-α mediated hyperpermeability and increased TEER in activated HBMVEC (Tang et al., 2018). Using this B^3^C microfluidic assay, we demonstrated a more critical role of PKCδ in regulating neutrophil transmigration as compared to neutrophil adhesion, providing important mechanistic insight into the mechanism of action of PKCδ. Our *in vitro* results agree with our *in vivo* studies in a rodent model of sepsis where PKCδ inhibition reduced BBB permeability as measured by Evans Blue uptake into the brain (Tang et al., 2018).

It is important to recognize that microphysiological systems, despite their utility in studying EC function, have inherent limitations. While they are often referred to as “organ-on-chip” systems, it is crucial not to interpret this term literally (Yang et al., 2021a). These systems typically involve the co-culture of a limited number of cell types within a predominantly mechanically rigid synthetic or gel scaffold. Consequently, they often lack the presence of other critical *in vivo* components, such as additional cell types, the extracellular matrix or other components. Experiments using microphysiological systems should therefore be carefully designed, and there is a need for cautious interpretation when extrapolating findings from these models to the more complex dynamics of *in vivo* systems (Yang et al., 2021b).

## Clinical research - sepsis heterogenicity and endotyping of patients

Devising a single standardized treatment for the heterogeneous sepsis patient population has proven problematic, emphasizing the recognition of the need to classify sepsis patients into distinct endotype classes that define specific host response subgroups (Leligdowicz and Matthay, 2019). Therefore, phenotyping sepsis patients in the clinical environment could identify patients for individualized treatment options.

Traditional methods to classify septic patients into distinct endotypes utilizes statistical models such as (k-means) clustering; most of these models suffer from lack of accuracy and/or validation (Li et al., 2022). For example, Li et al. collected 17 published clinical studies that focused on phenotyping adult septic patients (Li et al., 2022). However, only 6 out of 17 studies in the report were given a low risk of bias rating by the authors; the rest of the trials were deemed medium risk (8/17) and high risk of bias (3/17) due to lack of either internal validation or external validation or both (Li et al., 2022). This report implies that the traditional clustering models might not always yield reliable outcome to phenotype sepsis patients. In pediatric sepsis clinical research, Lin et al. suggested classifying new or progressive multiple organ dysfunction syndrome (NPMODS) during the 7-day observation as a distinct phenotype for better clinical outcomes (Lin et al., 2017). The authors report that the hospital mortality in patients with NPMODS was 51% compared with patients with new multiple organ dysfunction syndrome (28%) and those with single-organ dysfunction without multiple organ dysfunction syndrome (10%) (p < 0.001) (Lin et al., 2017). This study provides important clinical insight, but multi-institutional prospective clinical trials are needed for validation. There are many reports suggesting the use of biomarkers to phenotype patients For example, plasma lipoprotein level (Barker et al., 2021; Guirgis et al., 2021), coagulation markers (Barker et al., 2021; Kudo et al., 2021), hypoxia-inducible factor-1 (HIF-1) in macrophages (Peyssonnaux et al., 2007), source of contracting sepsis (Rosales et al., 2023) and vital signs such as hypotension or elevated lactate (Aldewereld et al., 2022) were used as criteria for phenotyping clusters. Most of these studies were done retrospectively using unsupervised clustering approaches. Although they provide important information for future research, prospective clinical trials are needed for validation. Advanced and more clinically relevant phenotyping mechanisms need to be developed for septic patients to receive customized treatment for better clinical outcomes.

Sepsis is an aggressive, heterogeneous disease that requires novel tools to characterize critical stages of the disease in each patient; omics analysis can help discover and characterize sepsis-related biomarkers (Langston et al., 2022). Specifically, omics can help in the identification of a) prognostic biomarkers, b) diagnostic biomarkers and c) biomarkers for personalized patient response to therapeutic intervention (Průcha et al., 2018; Langston et al., 2022). Since one biomarker is unlikely to represent a patient’s comprehensive sepsis condition, a synergistic panel of biomarkers that could satisfy the aforementioned criteria is needed (Průcha et al., 2018). A recent review analyzed 7 biomarkers that were most studied in sepsis: CRP, sTREM-1, LBP, Presepsin, CD64, PCT and IL-6. Results indicated that IL-6, PCT, LBP, sTREM-1 and presepsin (98) had the highest diagnostic accuracy (Teggert et al., 2020). However, limitations of current biomarkers include: prognostic and diagnostic accuracy, variability in concentration during the different stages of sepsis, lack of studies comparing biomarkers and no consensus on diagnostic cut-off values for analysis (Pierrakos et al., 2020; Teggert et al., 2020).

Several studies have classified sepsis patients into endotypes based on modeling methodologies and genome and transcriptome data (Wong et al., 2009; Davenport et al., 2016; Scicluna et al., 2017; Sweeney et al., 2018; Seymour et al., 2019). These studies have correlated the features of each endotype with clinical outcomes, demonstrating the heterogeneity of sepsis in patients. Since sepsis affects a myriad of biological compartments and cell types, omics can interrogate patient-specific genome, transcriptome and proteome expression in cells and tissues and, in combination with clinical and functional studies, decipher how biological pathways are dysregulated (Hasin et al., 2017; Langston et al., 2022). These findings will allow individual, molecular sub-typing of patients and distinguish the mechanisms in each endotype (Itenov et al., 2018; Olivier et al., 2019; Langston et al., 2022). A seminal genome-wide study identified three subclasses, via hierarchical clustering, in pediatric septic shock patients (Wong et al., 2009). Subclass A patients exhibited an immunocompromised presentation (due to repressed gene expression (e.g., Linker of activated T cells (LAT) and T-cell receptor-associated transmembrane adapter 1 (TRAT) in immunity) and thus had the highest mortality compared to subclasses B and C (Wong et al., 2009). Repressed genes were correlated with glucocorticoid and B-cell pathway signaling which further confirms that this group of patients did not have strong immunity (Wong et al., 2009). In a second study, the Molecular Diagnosis and Risk Stratification of Sepsis (MARS) project was designed to evaluate an 8 gene signature in classifying ICU sepsis patients into distinct endotypes (Scicluna et al., 2017). The endotypes generated from this study included MARS 1 (a group of patients showing decreased acquired and innate immunity gene expression), MARS 2 (patients exhibiting increased chemokine expression), MARS 3 (patients presenting increased acquired immunity expression) and MARS 4 (patients displaying increased NF-кB and interferon expression) (Scicluna et al., 2017). When developing a biomarker signature based on omics for distinguishing the heterogeneity of sepsis between patients, it should be simple and sensitive enough for patient classification to be feasible for use at the bedside (Langston et al., 2022). Furthermore, comparing endotype studies is complicated due to the differences in genome/transcriptome expression at various times (Langston et al., 2022). For example, most of the cited studies collected data in the first 48 hours from the time of hospital admission; however, it has been found that half of patients can change endotypes within the first 100 hours of admission (Leligdowicz and Matthay, 2019). Thus, omic expression needs to be carefully tracked as the disease progresses.

Even though the methodologies used to stratify patients into unique endotypes were different, there are similarities across endotypes which may be helpful for future therapeutic research (Langston et al., 2022). Low mortality endotypes [MARS 3 (Scicluna et al., 2017), subclasses B and C (Wong et al., 2009)] had increased immune signaling compared to high mortality endotypes [MARS 1 (Scicluna et al., 2017), subclass A (Wong et al., 2009)] which had repressed immune function (Langston et al., 2022). Overall, endotyping could be used to discover subphenotypes of a disease for tailoring of therapeutics, but it should not be used as a definitive, prognosis tool (Wong et al., 2017). Furthermore, additional, endotype studies are needed to examine their signatures across populations beyond their original intent and to observe if combinations of different endotypes can unravel novel, biological entities between populations leading to clinical presentation (Langston et al., 2022). However, before these advanced studies can be executed, there needs to be standardization by which endotypes are defined and standardized in various studies (Langston et al., 2022). Most importantly, endotype studies must be validated in prospective studies across institutions with sepsis patients who are at different stages of sepsis progression before they can be clinically relevant (Langston et al., 2022).

## *In silico* models – phenotyping using omic methodologies, machine learning and artificial intelligence

Because of the heterogenous nature of sepsis at omic, functional, clinical and temporal levels which results in phenotype differences (e.g., hypoinflammatory vs. hyperinflammatory phenotypes), data incorporated into *in silico* models are often collected and analyzed from diverse, reputable sources in order to comprehensively capture a patient’s condition. Most importantly, the integration of these sources of data into a model can expedite the drug discovery process and generate novel hypotheses that can be tested experimentally. *In silico* models offer a promising approach to integrate omics findings with functional measures from innovative tools like microphysiological systems and clinical parameters, enabling the generation of testable hypotheses and expediting the drug discovery process. These models also can unravel complex mechanisms of action that may not be readily apparent through traditional statistical analysis and/or reductive studies (Langston et al., 2022). Multiple modeling methods have been applied to sepsis that are quantitative (e.g., data-driven, multivariate regression, PCA) or qualitative (e.g., network) (Vodovotz and Billiar, 2013). Most models have focused on dysregulation of the immune response (Shi et al., 2015; Stan et al., 2018) but have not identified novel drugs or druggable targets that can be repurposed for sepsis. *In silico* modeling studies that not only incorporate large sets of data from different repositories but also focus on investigating new drugs or repurposing drugs for sepsis are urgently needed to address this life-threatening condition.

Drug repurposing or repositioning is an emerging area in drug discovery. The focus of the field is to identify novel uses for approved therapeutics that are outside the scope of the original disease (e.g., breast cancer) or indication (e.g., hematology) of interest (Pushpakom et al., 2018). The key advantage of this approach is the low risk of failure since the drug has already been approved for a specific disease/indication and has passed the extensive safety assessments. Furthermore, drug repurposing saves time and costs associated with moving a drug from the bench to the bedside, since on average, *de novo* drug development is a 10–17-year process with a cost of ~$2 billion. Moreover, the probability of a drug entering the market is below 10%; thus, alternative approaches are needed (Rumienczyk et al., 2022). Drug repurposing in sepsis has not yet reached its full potential, but efforts are underway; this is especially important since all ~150 drugs recently designed to treat sepsis have been successful in rodent models but have failed in clinical trials (Drake, 2013). This absence of clinical translation is due to a host of factors, including the incorporation of rodent models that fail at emulating the complete clinical situation that is present in humans (e.g., age, sex, demographics, comorbidities etc.) and the varied composition of systemic leukocytes in both species (humans have higher numbers of circulating neutrophils compared to mice) (Drake, 2013; Efron et al., 2015). These factors could significantly alter the trajectory of the disease (Langston et al., 2023). A recent review discussed the repurposing of oncology drugs to treat sepsis and identified several potential compounds (e.g., Topotecan, Olaparib, Trametinib) that are currently being evaluated in sepsis models (Rumienczyk et al., 2022). Another review identified several drugs (e.g., Methylthiouracil, Simvastatin, Mangiferin) used in endocrinology and oncology that are being repurposed as possible treatment approaches for sepsis (Prakash et al., 2020). However, there have not been many studies that use *in silico* modeling and drug repurposing to investigate neutrophil-EC dysregulation in sepsis.

We are using a synergistic combination of omics, *in silico* modeling and drug repurposing to investigate neutrophil-endothelial interaction in sepsis/inflammatory conditions. Recently, we implemented proteomic analysis of endothelial cells (from lung, liver and kidney) to investigate differences in protein expression levels between organ-specific ECs under inflammatory conditions across time. As time progressed, the number of differentially expressed proteins shared between ECs increased (Rossi et al., 2022). These findings suggest that sepsis progressively affects protein expression in different organ-specific ECs over time. Currently, we are identifying signaling pathways that have a mechanism of action associated with FDA approved drugs for repurposing. Using a combination of functional responses integrated with proteomic and genomic analysis, we will be able to evaluate the feasibility of repurposing these drugs for treating sepsis.

Machine learning (ML), a branch of Artificial intelligence (AI), can be used to improve sepsis diagnosis, prognosis and clinical/drug monitoring of the disease (Langston et al., 2023). For example, Goto et al. utilized a ML approach to identify sepsis patients with distinct phenotypes that could benefit from recombinant human thrombomodulin (rhTM) therapy (Goto et al., 2022). With this methodology, the authors developed a web-based application to identify rhTM target phenotype. Although promising, this model has not been validated in prospective studies (Vincent et al., 2019; Goto et al., 2022). ML has also been used to create a proteomic panel that distinguishes acute respiratory distress syndrome patients from sepsis patients and to predict early sepsis onset and prognosis (Yehya et al., 2022; Moor et al., 2021). However, there have not been any ML studies that specifically investigate neutrophil-endothelial interactions in sepsis and identify repurposed therapeutics which are urgently needed.

To advance the field, it is essential to explore the integration of genomic, transcriptomic, proteomic and metabolomic approaches, since multi-omics holds the potential for improved prognostication and the selection of more suitable treatments (von Groote and Meersch-Dini, 2022). However, the effectiveness of these approaches in improving clinical outcomes needs to be demonstrated through further rigorous clinical studies for validation (Arcaroli et al., 2005). Exciting advancements in omics technology offer promise in enhancing our understanding of disease pathophysiology at an individual patient level, thus facilitating the progression of precision medicine in sepsis and aiding in the development and validation of biomarkers (Davenport et al., 2016). Moreover, adopting more comprehensive tissue interrogation techniques, such as single-nuclei RNA sequencing and single-cell proteomics (Bennett et al., 2023), has the potential to augment biomarker-driven approaches by providing deeper insights into injury mechanisms and the localization of damage (Davenport et al., 2016). These strategies could provide the granularity required to implement a true precision medicine treatment approach for sepsis-related complications, such as acute kidney injury (AKI) (Kiryluk et al., 2018). Given the large volume of data generated from omics studies and the need to synergistically combine and validate this data with those obtained from microphysiological systems and clinical data, *in silico* models and ML will likely play a critical role in integrating these multiple types of data to allow for a comprehensive understanding of sepsis.

## Future directions

**Figure 4:**
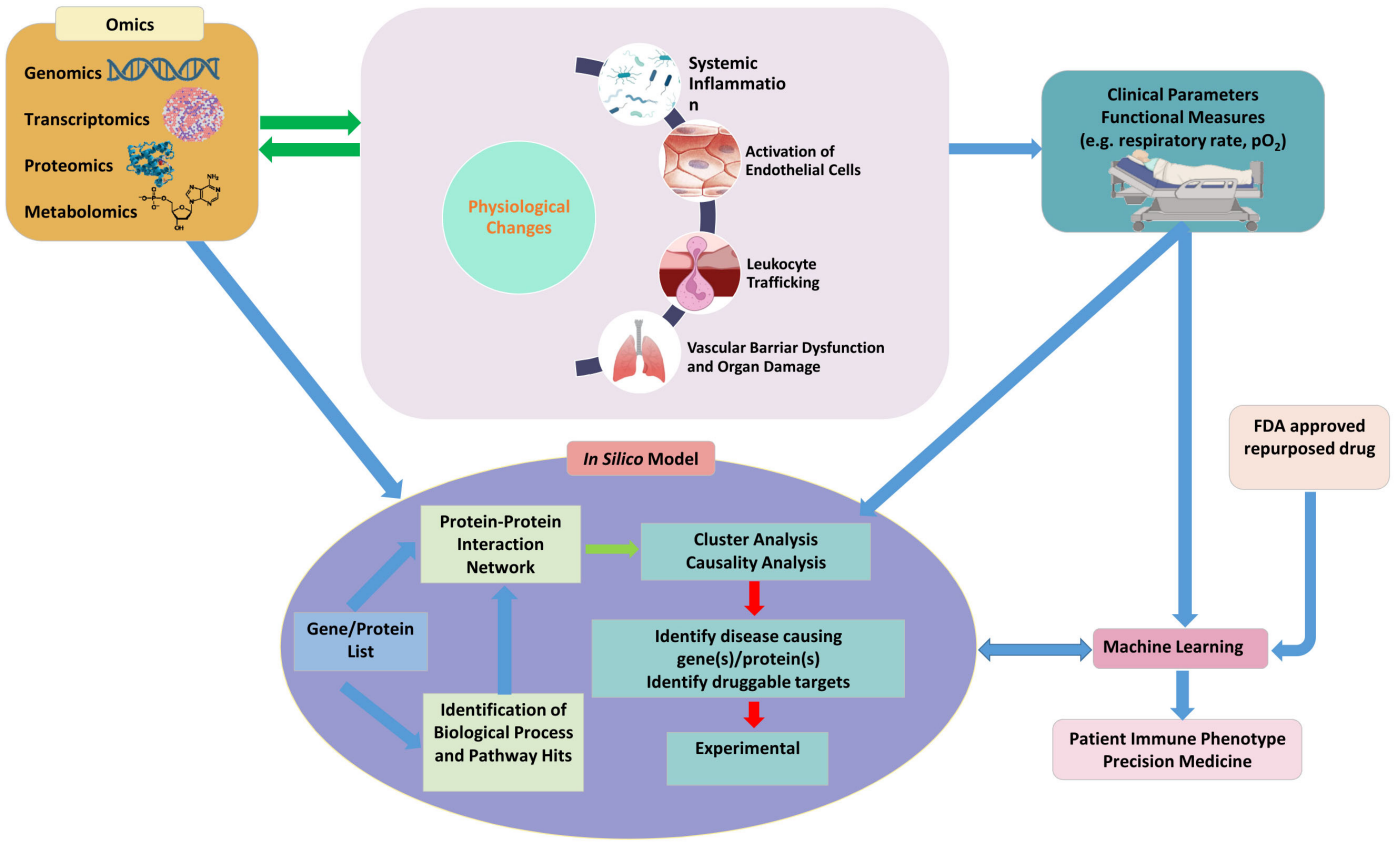
A synergistic platform incorporating emerging technologies such as microphysiological systems to study physiological function, clinical parameters, omics and *in silico* models combined with machine learning and artificial intelligence will not only provide a better understanding of the pathophysiology of sepsis but also outline a roadmap for precision medicine for treating sepsis.

In this review, we suggest that leveraging a multidisciplinary approach that incorporates innovative technologies such as microphysiological systems, translational clinical research, and *in silico* methods incorporating omics, machine learning and artificial intelligence (AI) in sepsis research will enhance our understanding of sepsis and bring about the promise of precisions medicine (**Figure 4**). Microphysiological systems offer a promising avenue for studying sepsis *in vitro* by mimicking the physiological conditions of the human body and providing a more accurate representation of the complex interactions between different cell types, tissues and the immune system. Future research studies should aim to optimize and standardize these platforms so that findings from different studies can be compared. This is particularly important as the recent FDA Modernization Act 2.0 now allows for such alternatives to animal testing to bring a drug to human trials (Wadman, 2023). Integration of biomimetic microfluidic assays with advanced imaging techniques and real-time monitoring systems can provide valuable insights into dynamic cellular and molecular changes during sepsis progression.

Complementing *in vitro* studies with *in vivo* clinical research remains crucial for translating laboratory findings into clinical applications. Clinical trials that involve large cohorts of sepsis patients can help validate the efficacy and safety of potential treatments. These studies should focus on identifying patient subgroups with distinct sepsis endotypes, allowing for targeted interventions and personalized treatment strategies. In addition, investigating the long-term outcomes of sepsis survivors and identifying potential biomarkers associated with prognosis and response to therapy will be essential.

Furthermore, synergistic incorporation and interpretation of large volumes of diverse data from multiple sources requires the use of emerging techniques such as *in silico* models and machine learning algorithms which can analyze and interpret vast amounts of data and assist in the prediction of disease progression, patient outcomes and treatment responses. These models can provide a holistic view of the dynamics of sepsis leading to a deeper understanding of its pathophysiology, facilitating the development of novel therapeutics which can ultimately improve patient care.

## Author contributions

DL: Visualization, Writing – original draft, Writing – review & editing. JL: Writing – original draft, Writing – review & editing. BP: Writing – original draft. MK: Conceptualization, Supervision, Validation, Writing – review & editing, Funding acquisition, Visualization. LK: Conceptualization, Funding acquisition, Supervision, Validation, Visualization, Writing – review & editing.

## Glossary

**Table:**

AI	Artificial intelligence
AKI	Acute kidney injury
B^3^C	Blood-brain-barrier on-a-chip
C5	Complement 5
CAMs	Cell adhesion molecules
CD64	Cluster of differentiation 64
CRP	C-reactive protein
CS	Chondroitin sulphate
CXCL8	C-X-C motif chemokine ligand 8
DAMPs	Damage-associated molecular patterns
DEGs	Differentially expressed genes
ECs	Endothelial cells
eGC	Endothelial glycocalyx
ESAM	Endothelial cell-selective adhesive molecules
EVs	Extracellular vesicles
FDA	Food and Drug Administration
fMLP	N-Formylmethionyl-leucyl-phenylalanine
GO BP	Gene Ontology Biological Process
H_2_O_2_	Hydrogen peroxide
HA	Hyaluronan
HBMVEC	Human brain microvascular endothelial cell
HLA	Major histocompatibility complex
HLA-DMA	Major histocompatibility complex, class II, DM alpha
HOCl	Hypochlorous acid
HS	Heparan sulphaste
Hypoxia-inducible factor-1	hypoxia-inducible factor-1
ICAM-1	Intercellular adhesion molecule 1
IFI6	Interferon alpha inducible protein 6
IFIT1	Interferon induced protein with tetracopeptide repeats 1
IFN-γ	Interferon-gamma
IL-15	Interleukin-15
IL-1β	Interleukin-1 beta
IL-6	Interleukin-6
IL-8	Interleukin-8
JAMs	Junctional adhesion molecule
LAT	Linker for activation of T cells
LBP	Lipopolysaccharide binding protein
MARS	Molecular Diagnosis and Risk Stratification of Sepsis
MCP-1	Monocyte chemoattractant protein 1
miRNA	Micro-RNAs
ML	Machine learning
MMP25	Matrix metalloproteinase 25
MMP8	Matrix metalloproteinase 8
MMP9	Matrix metalloproteinase 9
MODS	Multiple organ dysfunction syndrome
MPO	Myeloperoxidase
NADPH	Nicotinamide adenine dinucleotide phosphate
NETs	Neutrophil extracellular traps
NF-κB	Nuclear factor-kappa beta
NFKBIA	Nuclear factor kappa beta inhibitor alpha
NPMODS	New or progressive multiple organ dysfunction syndrome
O_2_^-^	Superoxide anion
OH^•^	Hydroxide free radical
PAMPs	Pathogen-associated molecular patterns
PCT	Procalcitonin
PD-L1	Programmed cell death ligand 1
PECAM-1	Platelet-endothelial cell adhesion molecule 1
PKCδ	Protein Kinase C-delta
PPI	Protein-protein interaction
PRRs	Pattern recognition receptors
rhTM	Recombinant human thrombomodulin therapy
RNA-seq	RNA-sequencing
ROS	Reactive oxygen species
SDC-1	Syndecan-1
sTREM-1	Soluble-triggering receptor expressed on myeloid cells-1
TNF-α	Tumor necrosis factor-alpha
TRAT	T-cell receptor-associated transmembrane adapter 1
VCAM-1	Vascular cell adhesion molecule 1
WGCNA	Weighted gene correlation network analysis
WHO	World Health Organization
